# Inhibitory Effects of Polyphenols-Rich Components From Three Edible Seaweeds on Inflammation and Colon Cancer *in vitro*

**DOI:** 10.3389/fnut.2022.856273

**Published:** 2022-05-13

**Authors:** Lingxiao Yi, Qi Wang, Haiyan Luo, Daqing Lei, Zhonghai Tang, Sijia Lei, Hang Xiao

**Affiliations:** ^1^Department of Food Science, University of Massachusetts, Amherst, MA, United States; ^2^School of Food and Drug, Shenzhen Polytechnic, Shenzhen, China; ^3^College of Food Science and Technology, Hunan Agricultural University, Changsha, China

**Keywords:** edible seaweeds, *Laminaria japonica*, *Ulva lactuca*, *Porphyra tenera*, polyphenols, anti-inflammation, anti-colon cancer

## Abstract

Polyphenols from edible seaweeds display various health benefits which have not been adequately studied. This study aimed to characterize the composition of extractable polyphenol-rich components (EPCs) and non-extractable polyphenol-rich components (NEPCs) from three edible seaweeds (i.e., *Laminaria japonica, Ulva lactuca*, and *Porphyra tenera*) and evaluate their anti-inflammatory capacities in activated macrophages and anticancer properties in colon cancer cells. Both EPCs and NEPCs from three edible seaweeds against lipopolysaccharides (LPS) stimulated nitric oxide in activated macrophages. Immunoblotting and qRT-PCR indicated that EPCs and NEPCs regulated the expression levels of proinflammatory enzymes, proinflammatory cytokines, and antioxidant enzymes in macrophages. Furthermore, EPCs and NEPCs lowered the viability of colon cancer cells, while normal colon cells were not affected. Additionally, EPCs and NEPCs induced cellular apoptosis and led to G0/G1 cell cycle arrest in HCT116 cells. Overall, these results provide a rationale for future animal and human studies designed to examine the anti-inflammatory and chemopreventive capacities of polyphenols-rich components from *L. japonica, U. lactuca*, and *P. tenera*.

## Graphical Abstract

Extractable polyphenols-rich components (EPCs) and non-extractable polyphenols-rich components (NEPCs) have been isolated from three edible seaweeds. EPCs and NEPCs from three edible seaweeds contain certain amounts of phenolics, flavonoids, and tannins, and few amounts of carbohydrates and proteins. Further studies evaluated the efficacy and mechanism behind the anti-inflammatory capacities in activated macrophages and anticancer properties in colon cancer cells. 
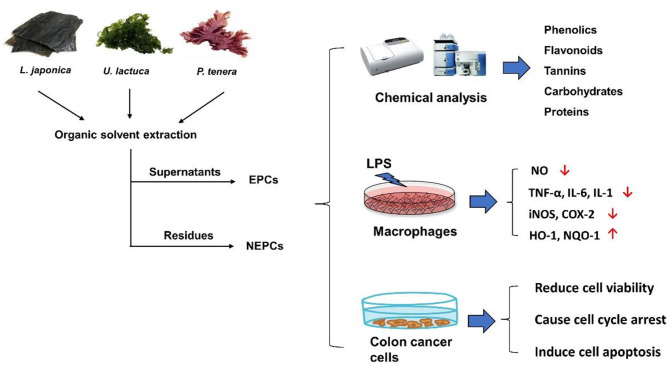

## Introduction

Polyphenols are secondary metabolites from plants which may offer health benefits against chronic diseases, such as oxidative stress, inflammation, and cancer (1). Polyphenols can be divided into two categories during the process of extraction, namely, extractable polyphenols that can be acquired by aqueous organic solvent and non-extractable polyphenols that remained in the residues and were largely ignored in most prior studies (2). Non-extractable polyphenols include low molecular weight polyphenols (phenolic acids and flavonoids) cross-linking with dietary fiber and proteins, and macromolecules polyphenols (condensed tannins and proanthocyanidins) (3). These phenolic compounds interact with the food matrix *via* hydrogen bonding, covalent bonding, and hydrophobic interactions (4). Moreover, non-extractable polyphenols with lower bioavailability in the stomach and small intestine reach the colon intact. Non-extractable polyphenols may release from the food matrix in the colon by the action of gut microbiota and then become bioactive and bioavailable (5). Additionally, non-extractable polyphenols compounds isolated from fruits, such as cranberry, strawberry, and apple, and vegetables have been reported with antioxidative, anti-inflammatory, and anti-cancer properties (6–9).

Inflammatory agent is an essential response to harmful stimuli caused by stress, infection, and injury and is characterized by symptoms such as heat, swelling, redness, and pain (10). Chronic inflammation has a strong association with chronic diseases, including cancer and heart disease (11). Macrophages stimulated by lipopolysaccharides (LPS) or interferon-gamma excessively secrete proinflammatory cytokines, including interleukin (IL)-1, IL-6, and tumor necrosis factor-α (TNF-α), which in turn induce the expression of proinflammatory enzymes, namely, cyclooxygenase-2 (COX-2) and inducible nitric oxide synthase (iNOS) (12). Studies have indicated that overexpression of these proinflammatory cytokines and enzymes is associated with tumor formation in the brain, breast, lung, colorectal, and prostate (13, 14). Natural bioactive compounds from terrestrial plants have been reported to offer beneficial effects against chronic disease. The application of seaweeds, the largest and most complex algae, as foodstuffs for human health traced back to several 100 years ago in Asian countries, due to the richness of bioactive compounds such as polysaccharides, polyphenols, minerals, fatty acids, bioactive peptides, and proteins (15). Phenolic compounds from seaweeds have been shown to be against inflammation and cancer in cell culture and animal studies (16–18).

To date, multiple phenolic compounds have been isolated and quantified from edible seaweeds and have been reported with various biological properties (16, 19, 20). These phenolic compounds belong to extractable polyphenols, where the potential health benefits of non-extractable polyphenols from these popular edible seaweeds remain unclear. However, polyphenol compounds from edible seaweeds, particularly for those with protective effects on inflammation and colon cancer, have not been adequately investigated. Brown seaweed *Laminaria japonica*, red seaweed *Porphyra tenra*, and green seaweed *Ulva lactuca* are three popular edible seaweeds, which are widely distributed in Asian countries and used as a drug in Traditional Chinese Medicine (21–23). Thus, this study aims to characterize the compositions of extractable polyphenol-rich components (EPCs) and non-extractable polyphenol-rich components (NEPCs) from *L. japonica, P. tenera*, and *U. lactuca* and to investigate their anti-colon cancer and anti-inflammatory efficacy and mechanisms.

## Materials and Methods

### Materials

Dried powders of *L. japonica* and *U. lactuca* were obtained from Wonderful LLC (Fuzhou, Fujian, China), and dried powder of *P. tenera* was purchased from PlantGift LLC (Haozhou, Anhui, China), in January 2020. The seaweed powders were stored at −20°C before use. 3-Hydrobenzoic acid, 4-hydrobenzoic acid, ferulic acid, iso-ferulic acid, sinapic acid, phloroglucinol, syringic acid, coumaric acid, rutin, hesperidin, luteolin, rosmamaric acid, apigenin, caffeic acid, gallic acid, chlorogenic acid, vanillic acid, myricetin, morin, quecertin, acacetin, kaempferol, catechin, epicatechin, gallo-catechin, epigallocatechin gallate, epigallocatechin, and epicatechin-gallate were ordered from Shyuanye (Shanghai, China). 3-(4, 5-dimethylthiazol-2-yl) 2, 5-diphenyltetrazolium bromide (MTT), 2,2'-Azobis (2-amidinopropane) dihydrochloride, potassium persulfate, propidium iodine (PI), and lipopolysaccharides (LPS) from *E. coli* O55:B5 were purchased from Sigma-Aldrich (Natick, MA, USA). Annexing V/PI double staining was obtained from Bio Vision (Mountain View, CA, USA). 1,1-Diphenyl-2-picrylhydrazyl (DPPH) free radical and 2.2′-azinobis (3-ethylbenzothiazoline-6-sulfonic acid ammonium salt) (ABTS) were purchased from TCI America (Portland, OR, USA). The antibodies of iNOS, COX-2, and HO-1 were ordered from Santa Cruz (Dallas, TX, USA), and the antibody of β-actin as the loading control was purchased from Sigma-Aldrich (Natick, MA, USA).

### Preparation of Polyphenols-Rich Components

The extraction of polyphenols-rich components was conducted based on our previous report with some modifications (7). Briefly, a mass of 25 g of the dried powders of edible seaweeds was blended with 500 ml of chilled 70% (v/v) acetone aqueous solution (1% acetic acid). The blend was subjected to ultrasound vibration for half hour, before spinning at 3,000 g for 10 min. The residues were subjected to the same procedure two times. After that, the supernatant was pooled, concentrated, and subjected to the extraction of EPCs, and the residues were collected for the extractions of NEPCs.

For the preparations of EPCs, the resulting supernatants were dissolved in the same volume of methanol. The highly lipophilic molecules were removed by the extraction of hexane. After that, the methanol layers were pooled and concentrated, followed by the extraction of ethyl acetate three times. Finally, the upper layer was pooled, dried, and stored at −20°C for further analysis.

For the preparations of NEPCs, the residues were blended with sodium hydroxide (2M) at 37°C for 2 h, where the containers were purged with nitrogen. Then, concentrated hydrochloric acid was added to terminate the reaction, before spinning at 4,000 g for 10 min. Subsequently, the supernatant was processed for the extraction of ethyl acetate. Finally, the upper phase was pooled, dried, and stored at −20°C for further analysis.

### Evaluation of Total Phenolics Contents, Flavonoids Contents, Tannins Contents, Carbohydrates Contents, and Proteins Content

Phenolics contents were measured by the Folin–Ciocalteu method according to a previous study (24). A volume of 20 μl of samples or gallic acid solutions (0 to 200 μg/ml) was added into a 96-well plate with 20 μL distilled water and 20 μl of Folin-Ciocalteu reagent. The plate was kept at room temperature for 10 min, before adding 140 μl of 7% sodium carbonate. Finally, the plate was kept at room temperature for another 90 min, followed by measuring absorbance at 760 nm using a spectrophotometer (BioTek Instrument, Inc. Winooski, VT, USA), and the results were presented as mg of gallic acid equivalents per hundred g seaweed powder (mg GAE/100 g seaweed powder).

Flavonoids contents were measured by the aluminum trichloride method according to a previous study (25). A volume of 20 μl of samples or catechin solutions (0 to 200 μg/ml) was added into a 96-well plate with 10 μl of 5% sodium nitrite and 100 μl of distilled water. First, the plate was kept at room temperature for 6 min before adding 20 μl of aluminum chloride. Then, the plate was incubated at room temperature for another 5 min before adding 50 μl of sodium hydroxide (1M). Finally, the absorbance was monitored at 510 nm using a spectrophotometer (BioTek Instrument, Inc. Winooski, VT, USA), and the results were presented as mg of catechin equivalents per hundred g seaweed powder (mg CE/100 g seaweed powder).

Tannins contents were measured by the vanillin-sulfuric acid method according to a previous study (26). A volume of 20 μl samples or catechin solutions (0 to 200 μg/ml) was added into a 96-well plate mixed with 90 μl of 30% concentrated sulfuric acid and 90 μl of 4% vanillin in methanol and was kept at room temperature for 5 min. Finally, the absorbance was read at 510 nm using a spectrophotometer (BioTek Instrument, Inc. Winooski, VT, USA), and the results were presented as mg of catechin equivalents per hundred g seaweed powder (mg CE/100 g seaweed powder).

Carbohydrates contents were assessed by the phenol-sulfuric acid method according to a previous study (27). A volume of 50 μl of samples or glucose solutions (0 to 200 μg/ml) was added into a 96-well plate, followed by adding 30 μl of 5% phenol and 150 μl of concentrated sulfuric acid rapidly. Finally, the plate was heated at 90°C for 5 min, followed by measuring the absorbance at 490 nm using a spectrophotometer (BioTek Instrument, Inc. Winooski, VT, USA), and the results were presented as mg of glucose equivalents per hundred g seaweed powder (mg GE/100 g seaweed powder).

Proteins contents were evaluated by the BCA method with minor modifications (28). Results were presented as mg of protein per hundred g seaweed powder (mg protein/100 g seaweed powder).

### Evaluation of Antioxidant Properties of EPCs and NEPCs

Oxygen radical absorbance capacity (ORAC) was assessed following a previous study with minor modifications (29). A volume of 20 μl samples or Trolox solutions (0 to 200 μM) was added into a 96-well plate with 40 μl of of fluorescencein solution (75 μM). The plate was gently shaken and stored at 37°C for 2 min, before adding 140 μl of 0.8 M 2,2'-Azobis (2-amidinopropane) dihydrochloride solution. Finally, the plate was subjected to a microplate fluorescence reader (BioTek Instrument, Inc., Winooski, VT, USA), excitation was measured at 485 nm, and emission was measured at 528 nm. This process continued for 2 h, and the absorbance was recorded at an interval of 2 min. Results were presented as μmol of Trolox equivalents per g extract (μmol TE/g extract).

The DPPH·scavenging capacity was determined following a previous study with minor modifications (30). A volume of 20 μl of samples or Trolox solutions was added into a 96-well plate mixed with 180 μl of 50 μM DPPH·ethanol solution. Finally, the plate was kept at room temperature for half-hour, followed by measuring absorbance at 517 nm by a spectrophotometer (BioTek Instrument, Inc. Winooski, VT, USA), and the results were presented as Trolox equivalent antioxidant capacity.

The ABTS + scavenging capacity was determined following a previous study with minor modifications (30). First, the ABTS working solution was prepared by the ratio of 7 mM ABTS solution to 2.45 mM potassium persulfate solution, which is 1:5. Subsequently, a volume of 10 μl of samples or Trolox solution was added into a 96-well plate with 200 μl of ABTS working solution. Finally, the plate was stored at room temperature, avoiding light for 7 min, followed by measuring absorbance at 734 nm using a spectrophotometer (BioTek Instrument, Inc. Winooski, VT, USA), and the results were presented as Trolox equivalent antioxidant capacity.

### Identification of Phenolic Compounds

High-resolution LC/MS was performed by an Ultimate 3000 UHPLC system coupled with an Orbitrap Fusion mass spectrometer (Thermo Scientific, Waltham, MA, USA) in the mass spectrometry core facility at the University of Massachusetts Amherst. Chromatography separation was carried out by the reverse-phase Kinetex XB-C18 column (100 mm × 4.6 mm, 2.6 μm, Phenomenex, Torrance, CA, USA). Meanwhile, the mobile phase is made up of 5% acetonitrile with 0.1% formic acid (solvent A) and 0.1% formic acid in 100% acetonitrile (solvent B). The initial mobile phase composition was 15% solvent B and linearly elevated to 100% solvent B within 3 min and maintained for 10 min. Then, the concentration of solvent B was linearly decreased to 15% with 0.01 min and maintained for 1.99 min. The flow rate was 400 μl/min, and the injection volume was 5 μl. Data were acquired in positive ESI mode using a spray voltage of 3,250 V, with sheath and aux gas set to 50 and 15, respectively, and vaporizer and tube temperature both set to 300°C. Data processing was accomplished using Xcalibur V4.2 (Thermo Scientific, Waltham, MA, USA).

### Cytotoxicity and Nitrite Oxide Assay of RAW 264.7 Cells

The cytotoxicity of EPCs and NEPCs on macrophages was tested according to the MTT assay, and the Griess test was carried out to investigate nitrite concentration as described earlier (31). RAW 264.7 cells (5 × 10^5^ cells/ml) were cultured into a 96-well plate (200 μl/well) and incubated for 24 h, before being treated with or without LPS (1 μg/ml) and coupled with an aliquot of EPCs or NEPCs at multiple concentrations for another 24 h. The cells and the culture media were subjected to MTT assay and Griess reaction, respectively.

### Cell Viability of Normal Colon Cells and Colon Cancer Cells

The cell viability was performed as reported in a previous study (32). CCD-18Co cells (50,000 cells/ml) and HCT116 cells (12,500 cells/ml) were cultured into a 96-well plate (200 μl/well) and incubated at 37°C overnight, before being posed to multiple concentrations of EPCs and NEPCs for another 48 h or 72 h. Finally, the cells were assessed by MTT assay.

### Flow Cytometer Analysis

Flow cytometer analysis was performed following previous studies (32, 33). HCT116 cells (4 × 10^4^ cells/ml) were cultured in 6-well plates and incubated overnight, before being posed to EPCs or NEPCs for 24 h for cell cycle analysis and for 48 h for cell apoptosis analysis. Then, media containing any floating cells were collected by trypsinization. Finally, cell pellets were washed by chilled PBS and subjected to flow cytometer analysis.

### qRT-PCR Analysis

Total RNA from macrophages was isolated by TRIzol reagent. The real-time qRT-PCR assay was carried out as reported in a previous study (31). The primer sequences used for cDNA amplification were listed in Supplementary Table S1. Three independent parallel groups were used, and related mRNA expression was determined using the 2^−Δ*ΔCt*^ method (34).

### Immunoblotting

The whole-cell protein extraction was based on a previous study (31). Briefly, macrophages were cultured in plates and incubated for 24 h, before being posed to multiple concentrations of EPCs or NEPCs with or without LPS for another 24 h. Then, the cell lysate was collected and assessed for immunoblotting following previous studies.

### Statistical Analysis

Data were presented as mean ± standard deviation (SD) of more than three independent parallel experiments. Statistical comparison among groups was performed using the one-way ANOVA followed by the student's *t*-test. A *p* < 0.05 was considered statistically significant.

## Results and Discussion

### Chemical Profiles of EPCs and NEPCs in *L. japonica, U. lactuca*, and *P. tenera*

Seaweed polyphenols have been reported to offer health benefits against oxidative stress, inflammation, and cancer (16, 18, 35). However, most studies into polyphenols focused only on the EPCs. NEPCs, the fraction of polyphenols remaining in the residue after extraction, were largely neglected by prior studies (2). In this study, we sought to elucidate the compositions of EPCs and NEPCs in three edible seaweeds, namely, *L. japonica, U. lactuca*, and *P. tenera*, and evaluate their potential protective effects on inflammation and colon cancer in this study.

**Figure 1:**
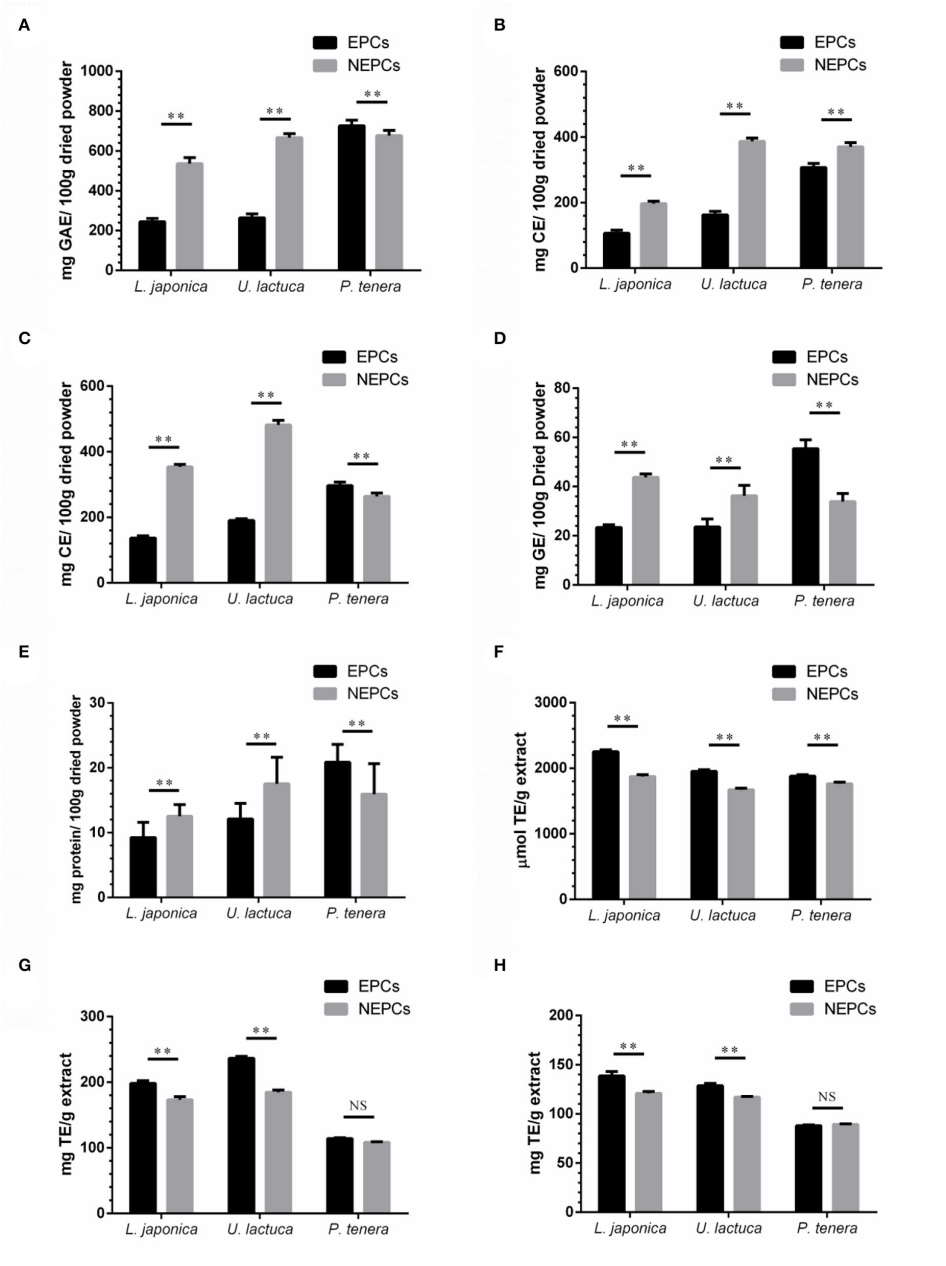
Total phenolic contents (PCs) **(A)**, flavonoid contents (FCs) **(B)**, tannin contents (TCs) **(C)**, carbohydrate content (CCs) **(D)**, and protein contents (PRCs) **(E)** in EPCs and NEPCs from three edible seaweeds. The levels of ORAC **(F)**, DPPH **(G)**, and ABTS **(H)** of the EPCs and NEPCs from three edible seaweeds. Data were presented as mean ± SD (*n* = 6). NS indicate no statistical difference, **p < 0.01 indicate a statistical difference.

The yield of EPCs from *L. japonica, U. lactuca*, and *P. tenera* was 9.85, 17.26, and 13.27 mg/g dried powder, respectively. The yield of NEPCs from *L. japonica, U. lactuca*, and *P. tenera* was 15.24, 17.75, and 19.45 mg/g dried powder, respectively. The total PCs, FCs, TCs, CCs, and PRCs in EPCs and NEPCs from the three edible seaweeds are shown in Figure 1. Interestingly, the PCs, FCs, and TCs in NEPCs from *L. japonica* were all higher than those in its EPCs. Similar patterns were observed in *U. lactuca*. Moreover, the PCs and TCs in EPCs were higher than those in its NEPCs in *P. tenera*. The FCs in NEPCs were also higher than those in EPCs in *P. tenera* (Figure 1). Small amounts of CCs and PRCs (<60 mg/100 g dried powder) were identified in EPCs and NEPCs. The relative abundance of PCs in NEPCs and EPCs was comparable with other plant-based foods, such as apples, bananas, carrots, broccoli, and lettuce (36). Also, both EPCs and NEPCs from *L. japonica* and *U. lactuca* contain more tannins than flavonoids.

The selected phenolic compounds in this study were identified and quantified by UHPLC/MS. Briefly, the abundance of phenolic compounds in the EPCs was higher than those in the NEPCs in the three edible seaweeds. But the amounts of phenolic compounds in *L. japonica, U. lactuca*, and *P. tenera* were different (Table 1). Hydrobenzoic acid, coumaric acid, chlorogenic acid, vanillic acid, caffeic acid, sinapic acid, quercetin, myricetin, catechin, epicatechin, and epigallocatechin gallate were the major constituents in the EPCs. In contrast, iso-ferulic acid, rosmarinic acid, luteolin, acacetin, and kaempferol were the major constituents in the NEPCs. Overall, this study offered an incisive understanding of the chemical profiles and biological effects of different bioactive components in *L. japonica, U. lactuca*, and *P. tenera*. More importantly, for the first time, we characterized the chemical profiles of their NEPCs.

**Table 1:** Selected phenolic compounds identified in the EPCs and NEPCs in *L. japonica, U. lactuca*, and *P. tenera*.

**Compounds**	**Retention time**	**MS(m/z)**	***L. japonica***		***U. lactuca***		***P. tenera***	
			**EPCS (μg/g extract)**	**NEPCs (μg/g extract)**	**EPCS (μg/g extract)**	**NEPCs (μg/g extract)**	**EPCS (μg/g extract)**	**NEPCs (μg/g extract)**
3-hydrobenzoic acid	3.96	139.039 ^+^	770.80 ± 48.92	26.66 ± 2.23	235.27 ± 14.25	23.02 ± 2.24	566.86 ± 50.25	133.70 ± 12.23
4-hydrobenzoic acid	3.15	139.039 ^+^	550.59 ± 33.52	ND	781.87 ± 59.82	ND	ND	ND
Phloroglucinol	2.91	127.039 ^+^	163.63 ± 15.21	ND	ND	211.60 ± 13.21	814.38 ± 78.82	433.62 ± 41.29
Sinapic acid	4.20	225.076 ^+^	530.84 ± 49.89	23.31 ± 2.04	506.95 ± 37.89	3.72 ± 0.92	742.58 ± 67.89	98.11 ± 8.02
Ferulic acid	4.25	195.065 ^+^	1175.76 ± 88.13	127.35 ± 2.65	16.32 ± 1.25	36.15 ± 2.52	211.24 ± 18.32	1020.73 ± 92.56
Iso ferulic acid	4.28	195.065 ^+^	ND	17.03 ± 1.52	ND	25.52 ± 1.78	89.69 ± 81.55	1256.29 ± 118.51
Syringic acid	4.33	199.060 ^+^	ND	ND	ND	ND	2726.17 ± 182.76	ND
Coumaric acid	3.70	165.055 ^+^	392.77 ± 4.44	ND	997.78 ± 93.42	ND	1436.86 ± 124.32	17.84 ± 1.84
Rosmarinic acid	4.20	361.092 ^+^	1446.66 ± 99.23	244.04 ± 19.21	ND	147.04 ± 12.23	815.44 ± 76.62	1027.65 ± 102.23
Chlorogenic acid	3.47	355.102 ^+^	1529.15 ± 111.13	ND	753.70 ± 61.53	191.01 ± 18.11	1928.50 ± 161.25	191.01 ± 18.11
Caffeic acid	3.16	181.050 ^+^	628.49 ± 41.22	ND	947.91 ± 81.24	189.36 ± 16.88	1323.60 ± 121.22	193.16 ± 17.89
Vanilic acid	3.15	169.050 ^+^	547.75 ± 51.23	8.82 ± 0.87	847.43 ± 31.63	ND	1325.73 ± 71.63	8.82 ± 0.87
Gallic acid	10.98	171.023 ^+^	2491.88 ± 216.21	ND	ND	ND	ND	ND
Luteolin	4.52	287.055 ^+^	2.39 ± 0.34	221.53 ± 16.54	411.52 ± 39.85	949.12 ± 86.54	408.16 ± 40.12	1074.05 ± 96.54
Rutin	3.88	611.161 ^+^	ND	ND	4033.13 ± 378.18	ND	3752.20 ± 368.28	ND
Hesperidin	4.00	611.197 ^+^	ND	ND	3120.40 ± 202.13	72.46 ± 6.85	2250.90 ± 202.13	104.55 ± 9.85
Myricetin	4.30	319.045 ^+^	897.06 ± 68.89	9.82 ± 0.75	724.98 ± 58.89	1.23 ± 0.07	422.91 ± 28.81	2.82 ± 1.75
Apigenin	4.77	271.060 ^+^	100.20 ± 9.13	15.65 ± 1.98	85.12 ± 6.78	33.31 ± 2.98	362.73 ± 26.78	92.51 ± 6.98
Morin	4.44	303.050 ^+^	44.30 ± 3.55	5.02 ± 0.51	405.14 ± 3.55	3.42 ± 2.12	1353.32 ± 123.52	1.99 ± 0.12
Quecertin	4.56	303.050 ^+^	937.03 ± 92.23	16.99 ± 1.96	1842.13 ± 89.23	5.99 ± 0.96	481.89 ± 29.35	8.99 ± 0.96
Acacetin	5.43	285.076 ^+^	ND	909.85 ± 92.97	ND	110.51 ± 9.24	4.91 ± 0.32	201.82 ± 19.24
Kaempferol	4.52	287.056 ^+^	3915.07 ± 256.11	213.06 ± 2.55	477.52 ± 36.11	929.76 ± 8.55	ND	988.04 ± 78.34
Catechin	3.70	291.086 ^+^	913.19 ± 82.23	1.12 ± 0.03	1926.78 ± 119.23	1.52 ± 0.43	2528.84 ± 231.23	6.22 ± 0.43
Epicatechin	3.86	291.086 ^+^	1263.01 ± 102.13	1.78 ± 0.76	737.84 ± 72.13	2.58 ± 0.76	1342.42 ± 132.13	12.58 ± 2.76
Gallocatechin	2.97	307.081 ^+^	854.06 ± 71.23	1.03 ± 0.16	1348.26 ± 71.23	ND	892.31 ± 16.35	ND
Epigallocatechin gallate	3.91	459.092 ^+^	1525.35 ± 109.12	132.26 ± 11.91	3357.55 ± 209.12	1195.54 ± 101.91	28475.45 ± 254.11	ND
Epigallocatehin	3.15	307.081 ^+^	1111.54 ± 96.15	ND	2016.63 ± 154.15	ND	2830.86 ± 211.81	8.18 ± 0.75
Epicatechin gallate	4.06	443.097 ^+^	ND	28.26 ± 1.95	397.85 ± 29.12	100.01 ± 12.95	2144.65 ± 121.53	136.59 ± 12.95

*Results were expressed as mean ± SD. ND means not detected*.

### Antioxidant Capacities of the EPCs and NEPCs in *L. japonica, U. lactuca*, and *P. tenera*

The abundance of phenolics, flavonoids, and tannins in EPCs and NEPCs of these seaweeds may have contributed to the antioxidant capacities. The ORAC values were ranging from 1,870 to 2,280 μmol TE/g in EPCs and from 1,750 to 1,880 μmol TE/g in NEPCs. Furthermore, the ORAC values of both EPCs and NEPCs from *L. japonica* were higher than the *U. lactuca* and *P. tenera* (Figure 1F). The DPPH values were ranging from 110 to 240 mg TE/g in EPCs and from 100 to 190 mg TE/g in NEPCs (Figure 1G). The ABTS values were ranging from 85 to 140 mg TE/g in EPCs and from 87 to 122 mg TE/g in NEPCs (Figure 1H). The antioxidant activity of EPCs from *L. japonica* and *U. lactuca* was all significantly higher than the NEPCs measured by the DPPH method and the ABTS method. There was no difference between the activity of EPCs and NEPCs from *P. tenera*. Overall, EPCs and NEPCs from *L. japonica* and *U. lactuca* exhibited stronger antioxidant activities than those from *P. tenera*.

### EPCs and NEPCs Reduced the NO Production in Activated Macrophages

Epidemiological data have revealed that a higher intake of polyphenols might reduce the risk of inflammation (37). Then, we sought to understand the protective effects of EPCs and NEPCs from the three edible seaweeds against inflammation in LPS-treated macrophages. First, their cytotoxicity on RAW264.7 macrophages was monitored by MTT assay at multiple concentrations. Both EPCs and NEPCs from the three edible seaweeds did not display any cytotoxicity up to 200 μg/ml (Supplementary Figure S1). Subsequently, these nontoxic ranges were used to evaluate their anti-inflammatory effects on activated macrophages.

**Figure 2:**
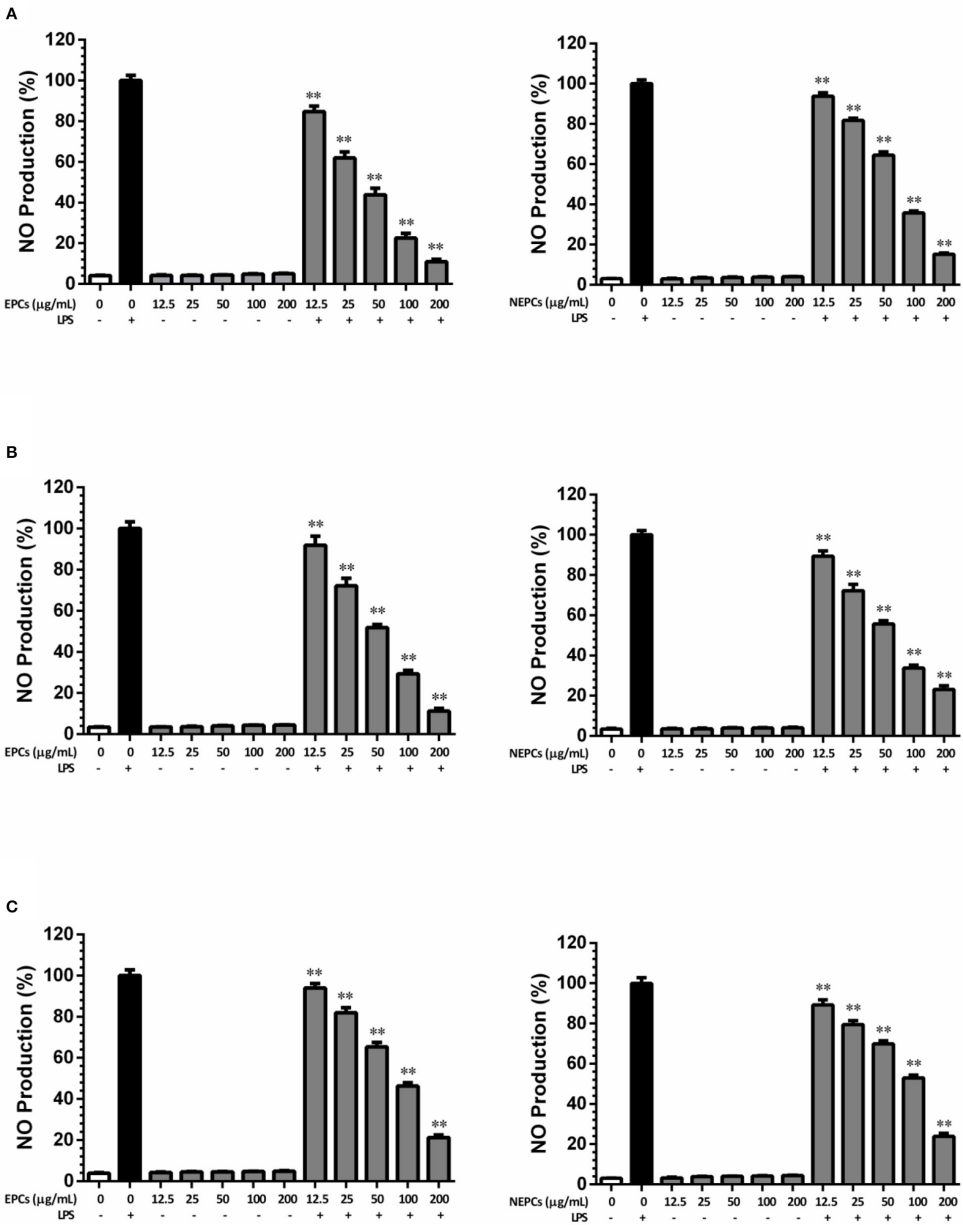
Inhibitory effects of EPCs and NEPCs from *L. japonica* **(A)**, *U. lactuca* **(B)**, *P. tenera* **(C)** on NO production in activated macrophages. Results were expressed as mean ± SD (*n* = 6). *p < 0. 05 and **p < 0.01 indicates statistically differences from LPS-treated group.

Nitrite oxide (NO) is a signaling molecule, and overproduction of NO during the inflammation process can induce proinflammatory cytokines in macrophages (38). In this study, LPS alone significantly stimulated NO production, when compared with the control group. Without LPS stimulation, EPCs or NEPCs from the three edible seaweeds did not trigger the overproduction of NO, while they significantly decreased the overproduction of NO stimulated by LPS in a dose-dependent manner. More specifically, the IC_50_ values of EPCs from *L. japonica, U. lactuca*, and *P. tenera* were 39.98, 52.43, and 82.43 μg/ml, respectively (Figures 2A–C). The IC_50_ values of NEPCs from *L. japonica, U. lactuca*, and *P. tenera* were 69.59, 60.83, and 93.54 μg/ml, respectively (Figures 2A–C). Overall, EPCs had stronger inhibitory effects on NO production in activated macrophages than NEPCs. Also, *L. japonica* and *U. lactuca* showed a stronger suppression for the production of NO than *P. tenera*.

### EPCs and NEPCs Lowered the Gene Expression of Proinflammatory Cytokines

**Figure 3:**
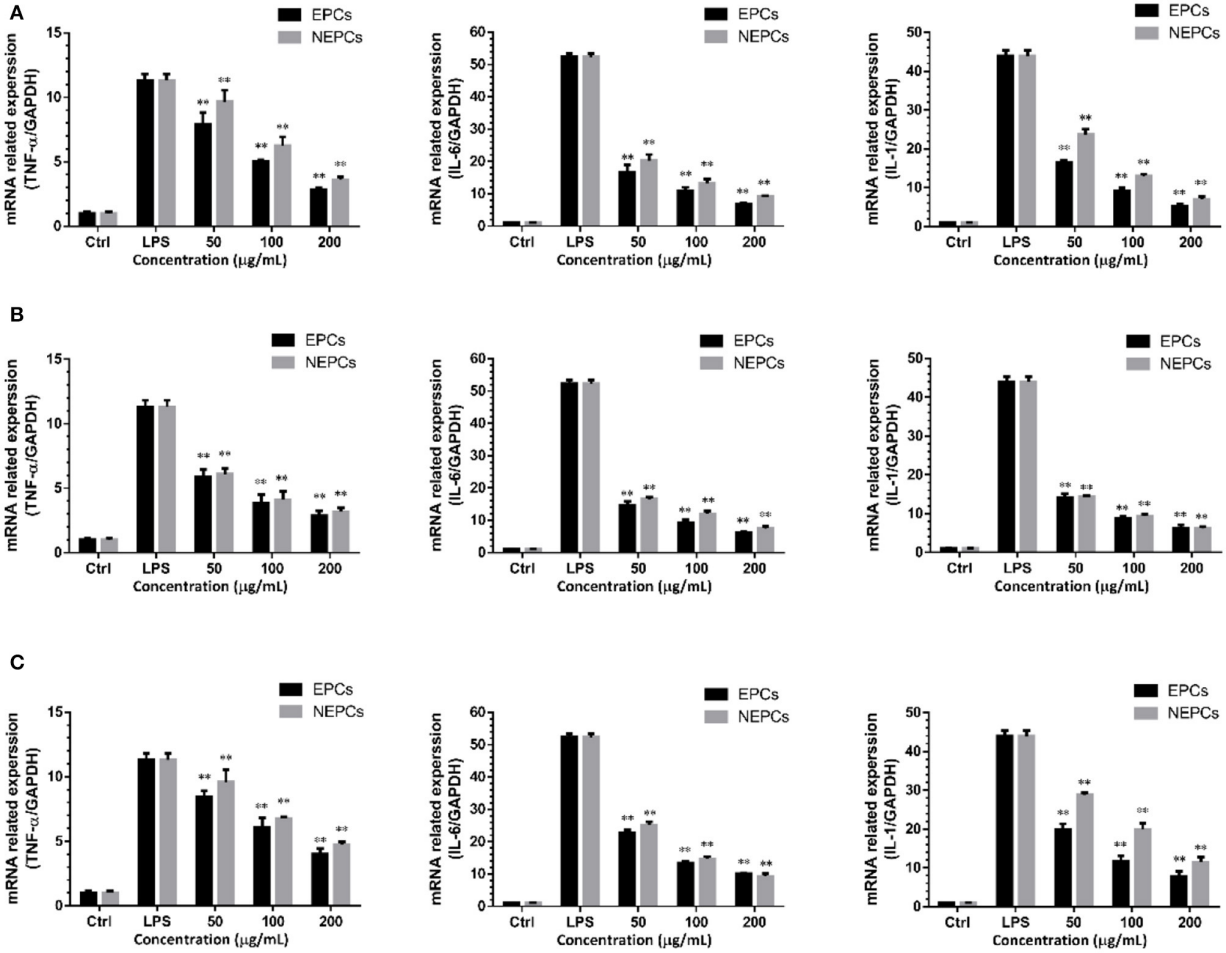
Suppressive effects of EPCs and NEPCs from *L. japonica* **(A)**, *U. lactuca* **(B)**, *P. tenera* **(C)** on mRNA expression of TNF-α, IL-6, IL-1 in activated macrophages. Results were expressed as mean ± SD (*n* = 6). *p < 0. 05 and **p < 0.01 indicate statistically differences from LPS-treated group.

The LPS stimulation also activates the macrophages to generate proinflammatory cytokines (39). The mRNA expression levels of IL-1, IL-6, and TNF-α were all slightly raised in response to LPS treatment, and these elevated cytokines were diminished by the treatment of EPCs or NEPCs (Figure 3). EPCs from *L. japonica, U. lactuca*, and *P. tene*ra, at 200 μg/ml, suppressed the mRNA expression levels of TNF-α by 74.86, 74.69, and 64.69%, respectively. NEPCs from *L. japonica, U. lactuca*, and *P. tenera*, at 200 μg/ml, reduced the mRNA expression levels of TNF-α by 68.14, 71.85, and 57.96%, respectively (Figures 3A–C). Moreover, similar patterns were observed in the mRNA expression levels of IL-6 and IL-1 (Figure 3). Our results indicated that both EPCs and NEPCs exerted anti-inflammatory effects *via* suppressing the overproduction of the aforementioned cytokines.

### EPCs and NEPCs Suppressed INOS and COX-2 Expression in Activated Macrophages

**Figure 4:**
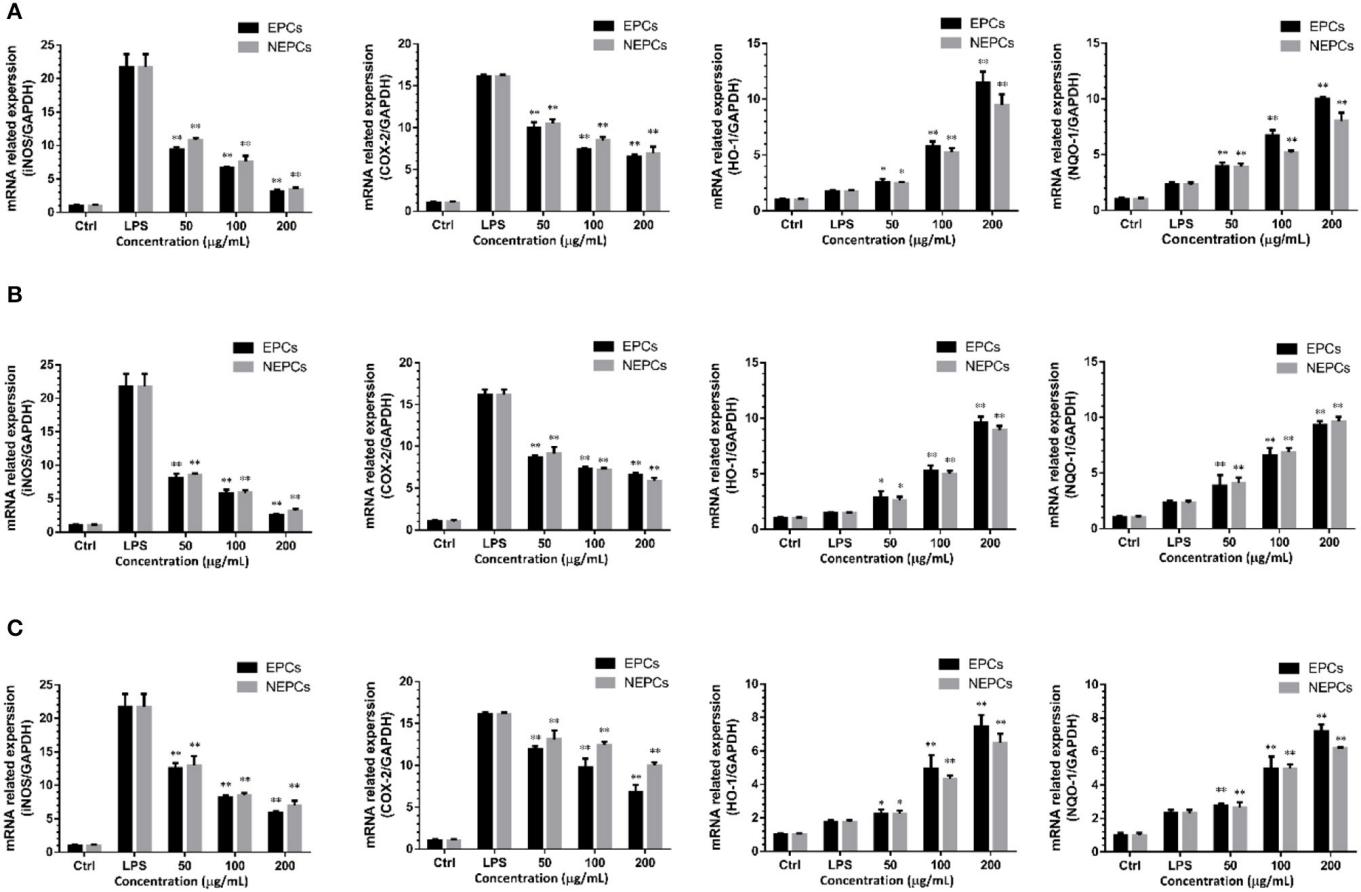
Effects of EPCs and NEPCs from *L. japonica* **(A)**, *U. lactuca* **(B)**, *P. tenera* **(C)** on mRNA expression of iNOS, COX-2, HO-1 and NQO-1 in activated macrophages. Results were expressed as mean ± SD (*n* = 3), *p < 0. 05 and **p < 0.01 indicate statistically differences from LPS-treated group.

**Figure 5:**
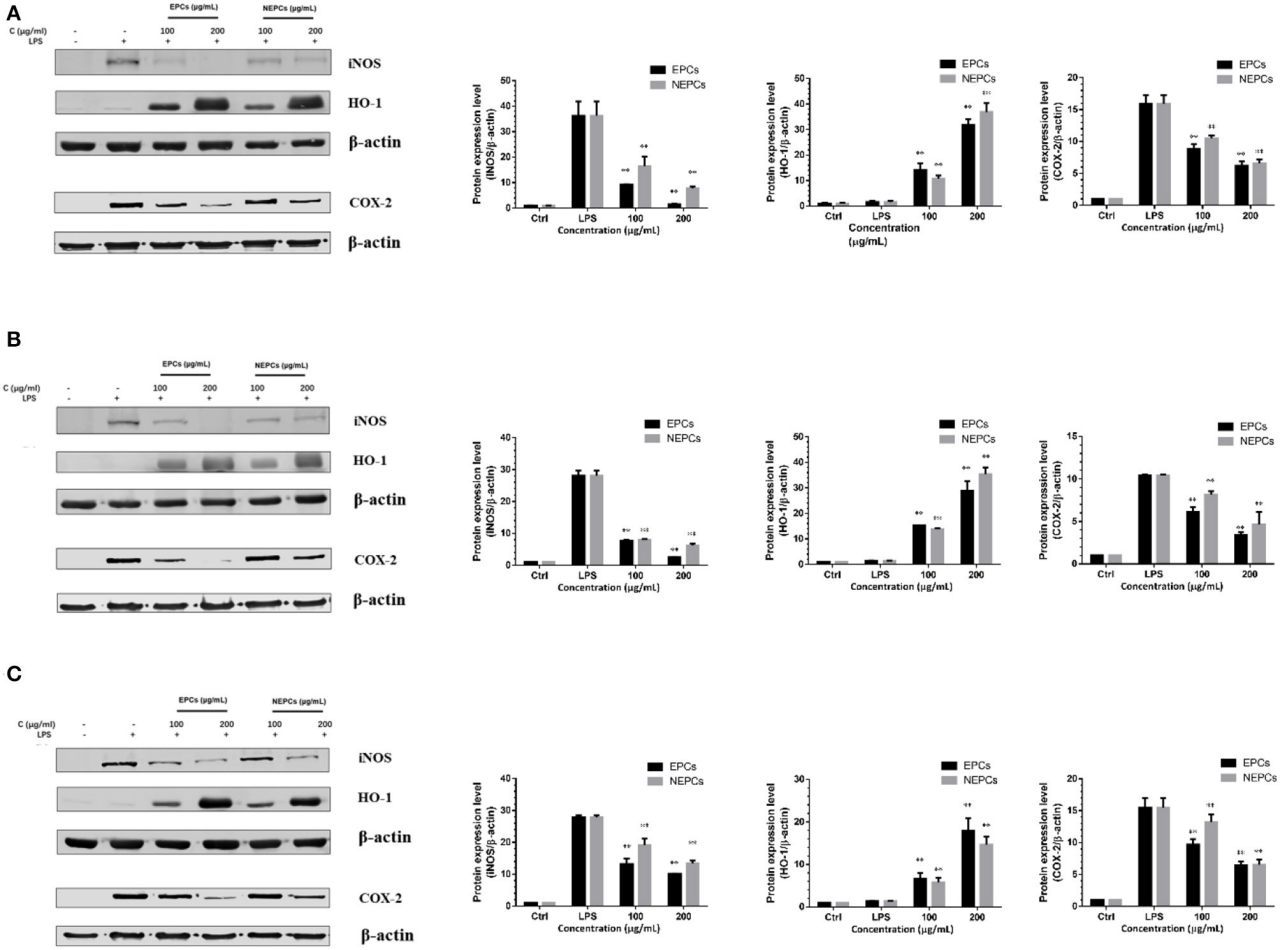
Effects of EPCs and NEPCs from *L. japonica* **(A)**, *U. lactuca* **(B)**, *P. tenera* **(C)** on protein expression of iNOS, COX-2 and HO-1 in activated macrophages. Results were expressed as mean ± SD (*n* = 3), *p < 0. 05 and **p < 0.01 indicate statistically differences from LPS-treated group.

Proinflammatory enzymes, especially for the COX-2 and iNOS, play a vital role in inflammatory response (40). The expressions of iNOS and COX-2 were greatly elevated in response to LPS stimulation. EPCs and NEPCs from the *three* edible seaweeds lowered their expression (Figures 4, 5). Specifically, EPCs from *L. japonica, U. lactuca*, and *P. tenera*, at 200 μg/ml, suppressed the mRNA expression levels of iNOS by 85.89, 88.47, and 72.99%, respectively. NEPCs from *L. japonica, U. lactuca*, and *P. tenera*, at 200 μg/ml, inhibited the mRNA expression levels of iNOS by 83.96, 85.11, and 67.92%, respectively (Figures 4A–C). Furthermore, the effects of EPCs and NEPCs from the three edible seaweeds on the protein expression of iNOS were similar to the mRNA expression levels. EPCs and NEPCs from the three edible seaweeds, at 200 μg/ml, reduced the protein expression of iNOS ranging from 52.27 to 95.74% (Figures 5A–C). Similar patterns were acquired in the expression of COX-2 (Figures 4, 5). These findings suggested that EPCs and NEPCs lowered the production of NO by downregulating iNOS and COX-2 signaling pathways.

### EPCs and NEPCs Elevated the Expression Levels of Antioxidant Enzymes in Activated Macrophages

Elevated expressions of HO-1 and NQO-1, two antioxidant enzymes, have been reported to reduce the overproduction of inflammatory enzymes and proinflammatory cytokines (41). As shown in Figure 4, EPCs and NEPCs from *L. japonica, U. lactuca*, and *P. tenera* significantly elevated the mRNA expression level of HO-1 and NQO-1, when compared with the LPS group. Specifically, EPCs from *L. japonica, U. lactuca*, and *P. tenera*, at 200 μg/ml, potently elevated the mRNA expression of HO-1 by 6.59-, 5.50-, and 5.59-fold, respectively. NEPCs from *L. japonica, U. lactuca*, and *P. tenera* enhanced the HO-1 mRNA expression by 5.45-, 5.02-, and 4.43-fold, respectively (Figures 4A–C). Similar patterns were obtained in the mRNA expression of NQO-1 (Figures 4A–C). In addition to the mRNA expression, EPCs and NEPCs from the three edible seaweeds also greatly upregulated the HO-1 protein expression, and their results were consistent with the qRT-PCR results (Figures 5A–C).

### EPCs and NEPCs Suppressed the Viability of Colon Cancer Cells

**Figure 6:**
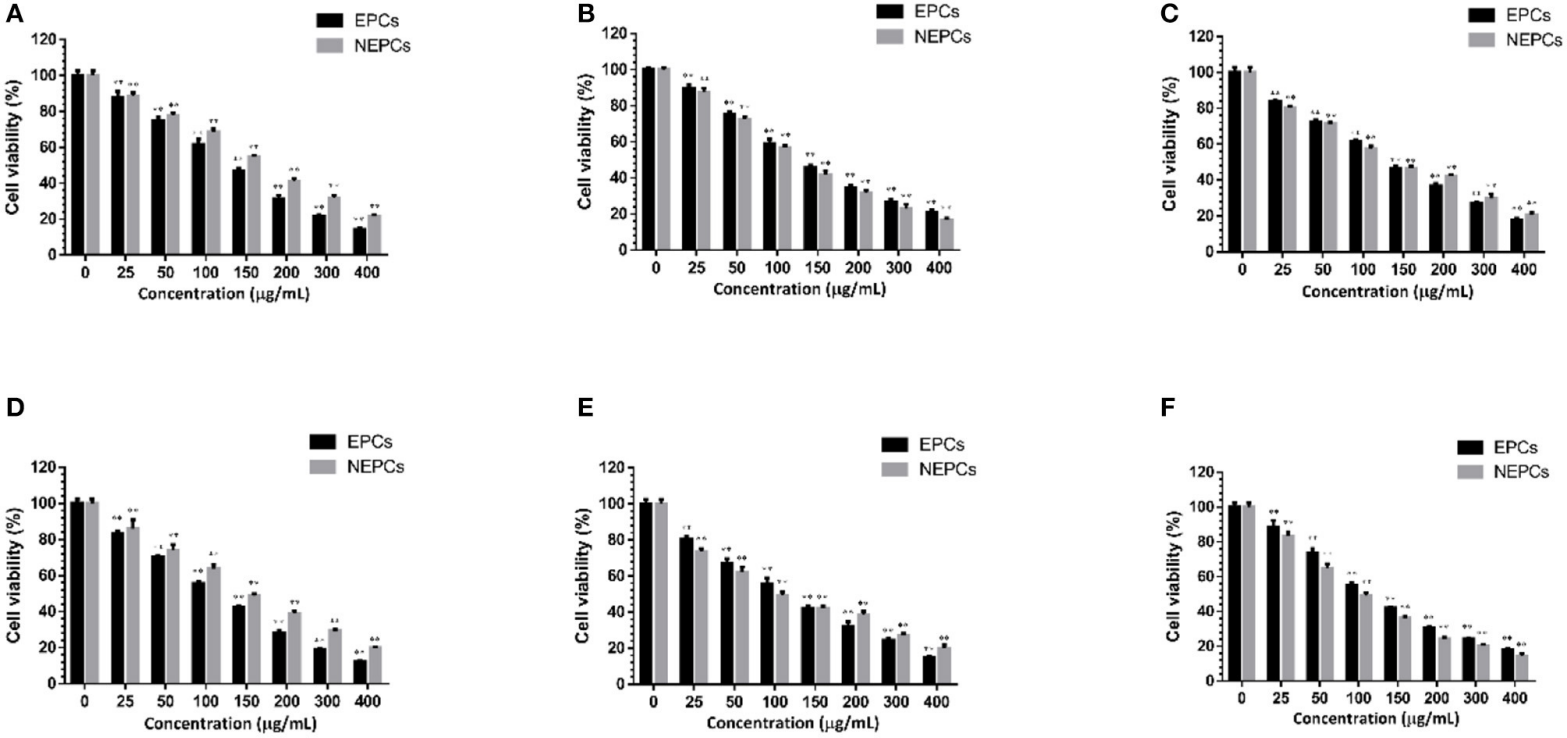
Suppressive effects of the EPCs and NEPCs from *L. japonica* **(A)**, *P. tenera* **(B)** and *U. lactuca* **(C)** on HCT116 cells for 48 hours; Suppressive effects of the EPCs and NEPCs from *L. japonica* **(D)**, *P. tenera* **(E)** and *U. lactuca* **(F)** on HCT116 cells for 72 hours. Results were presented as mean ± SD (*n* = 6). **p < 0.01 indicate statistically differences from untreated group.

A large number of phytochemicals with anti-inflammatory and antioxidant capacities also display protective effects on colon cancer (42). We found that EPCs and NEPCs from the three edible seaweeds did not cause any suppressive effects on the CCD18-Co cells up to 400 μg/ml for 72 h (Supplementary Figure S2). Thus, these concentrations were used to evaluate the anti-colon cancer effects in HCT116 cells. Furthermore, we found that EPCs and NEPCs from three edible seaweeds greatly lowered the cell viability of HCT116 cells in a time- and dose-dependent manner. Specifically, the IC_50_ values of EPCs from *L. japonica, U. lactuca*, and *P. tenera* after 48 h treatment were 124.2, 129.5, and 127.2 μg/ml, respectively. The IC_50_ values of NEPCs from *L. japonica, U. lactuca*, and *P. tenera* after 48 h treatment were 160.4, 130.5, and 127.5 μg/ml, respectively (Figures 6A–C). Moreover, the IC_50_ values of EPCs from *L. japonica, U. lactuca*, and *P. tenera* after 72 h treatment were 105.2, 115.6, and 104.9 μg/ml, respectively. In addition, the IC_50_ values of NEPCs from *L. japonica, U. lactuca*, and *P. tenera* after 72 h treatment were 139.3, 95.7, and 94.9 μg/ml, respectively (Figures 6D–F). Our results indicated that EPCs and NEPCs potently suppressed the viability of colon cancer cells, while normal colon cells were not affected at much higher concentrations.

### EPCs and NEPCs Led Cell Cycle Arrest and Apoptosis

**Figure 7:**
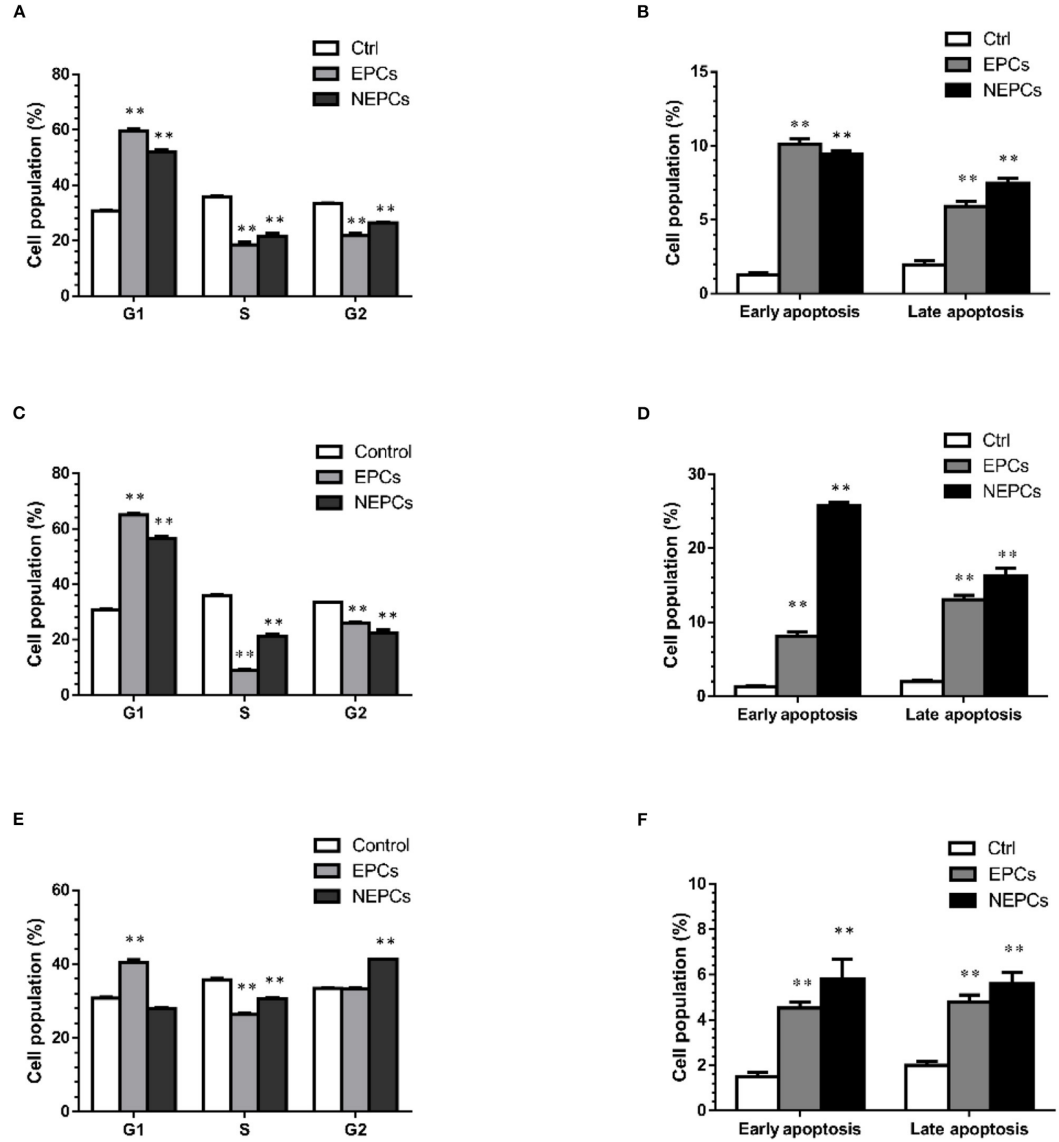
Quantification of cell cycle distribution posed to EPCs and NEPCs from *L. japonica* **(A)**, *U. lactuca* **(C)** and *P. tenera* **(E)**. Quantification of early and late apoptosis posed to EPCs and NEPCs from *L. japonica* **(B)**, *U. lactuca* **(D)**, *P. tenera* **(F)**. Results were presented as mean ± SD (*n* = 3). **p < 0.01 indicate statistically differences from untreated group.

Cell proliferation and apoptosis are two important therapeutic targets for cancer (43). In this study, we selected EPCs and NEPCs at the dose of 150 μg/ml for flow cytometry analysis. EPCs and NEPCs from *L. japonica* and *U. lactuca* noticeably elevated the cell accumulation in the G0/G1 phase. EPCs and NEPCs from *L. japonica* elevated the populations of HCT116 cells in the G0/G1 phase by 1.93- and 1.68-fold, respectively (Figure 7A, Supplementary Figure S3). Similar patterns were acquired in the analysis of the effects of EPCs and NEPCs from *U. lactuca* on the cell cycle distribution (Figure 7C, Supplementary Figure S3). Moreover, EPCs from *P. tenera* enhanced the populations of HCT116 cells in the G0/G1 phase by 31.14%, and NEPCs from *P. tenera* elevated the populations of HCT116 cells in the G2/S phase by 23.50% (Figure 7E, Supplementary Figure S3). For cell apoptosis analysis, EPCs and NEPCs greatly enhanced the apoptotic cell population. Specifically, EPCs from *L. japonica* raised cell population in the early and late apoptosis by 7.76- and 7.25-fold, respectively. NEPCs from *L. japonica*, increased cell population in the early and late apoptosis by 3.01- and 3.80-fold, respectively (Figure 7B, Supplementary Figure S4). Finally, the patterns of EPCs and NEPCs from *U. lactuca* and *P. tenera* were consistent with those of EPCs and NEPCs from *L. japonica* (Figures 7D,F, Supplementary Figure S4). These findings indicated that EPCs and NEPCs from the three edible seaweeds inhibited the growth of human colon cancer cells *via* the activation of cell cycle arrest and cellular apoptosis.

## Conclusion

These results, for the first time, elucidated the composition of polyphenols-rich components from the three edible seaweeds, *L. japonica, P. tenera*, and *U. lactuca*, and we further investigated their efficacy and mechanisms against inflammation and colon cancer in cell studies. We found that EPCs and NEPCs exerted potent inhibitory effects in activated macrophages *via* suppressing proinflammatory cytokines and enzymes and activating antioxidant enzymes. At the same time, they lowered the proliferation of HCT116 cells by inducing cell cycle arrest and cell apoptosis. The novel extracts of edible seaweeds may offer a safe, inexpensive, and efficacious dietary strategy to prevent colon cancer in humans, especially in individuals with chronic inflammation. Further work will comprehensively evaluate the anti-inflammatory capacities and anticancer properties of polyphenols-rich components from edible seaweeds in animal and human studies.

## Data Availability Statement

The original contributions presented in the study are included in the article/Supplementary Materials, further inquiries can be directed to the corresponding authors.

## Author Contributions

LY: methodology, experiment performance, and writing—original draft preparation. HX: writing—reviewing and editing. SL and HX: conceptualization. SL, HX, and ZT: supervision. DL: manuscript revision. HL: sample preparation. QW and LY: data collection and analysis. All authors contributed to the article and approved the submitted verison.

## Funding

This work was partially supported by the Shenzhen Strategic Emerging Industry Development Special Fund Project (Shenzhen Municipal Economic and Trade Information Commission, #20180130150742764 to SL), United States Department of Agriculture (MAS00450, MAS00492, NIFA grant #2019-67017-29249 and 2020-67017-30835 to HX), and Hunan Science and Technology Plan Program and Major Research Plan of Changsha (2019RS1055 and kq1801016 to ZT).

## Conflict of Interest

The authors declare that the research was conducted in the absence of any commercial or financial relationships that could be construed as a potential conflict of interest.

## Publisher's Note

All claims expressed in this article are solely those of the authors and do not necessarily represent those of their affiliated organizations, or those of the publisher, the editors and the reviewers. Any product that may be evaluated in this article, or claim that may be made by its manufacturer, is not guaranteed or endorsed by the publisher.

## Abbreviations

EPCs: extractable polyphenols-rich components
NEPCs: non-extractable polyphenols-rich components
LPS: lipopolysaccharides
NO: nitrite oxide
iNOS: inducible nitric oxide synthase
COX-2: cyclooxygenase-2
HO-1: heme oxygenase 1
NQO-1: NADPH-quinone oxidoreductase-1
TNF- α: tumor necrosis factors-α
IL: interleukin
PCs: phenolics contents
FCs: flavonoids content
TCs: tannins contents
CCs: carbohydrates contents
PRCs: proteins contents
ORAC: oxygen radical absorbance capacity.
